# Rapid Phenotypic Antimicrobial Susceptibility Testing Using a Coulter Counter and Proliferation Rate Discrepancy

**DOI:** 10.1021/acsomega.3c00947

**Published:** 2023-04-19

**Authors:** Qiaolian Yi, Jing Cui, Meng Xiao, Ming-Zhong Tang, Hui-Cui Zhang, Ge Zhang, Wen-Hang Yang, Ying-Chun Xu

**Affiliations:** † Department of Laboratory Medicine, Peking Union Medical College Hospital, Chinese Academy of Medical Science and Peking Union Medical College, Beijing 100730, China; ‡ Beijing Key Laboratory for Mechanisms Research and Precision Diagnosis of Invasive Fungal Diseases, Peking Union Medical College Hospital, Chinese Academy of Medical Science and Peking Union Medical College, Beijing 100730, China; § Scenker Biological Technology Co., Ltd, Liaocheng, Shandong 252200, China; ∥ State Key Laboratory of Complex Severe and Rare Diseases, Peking Union Medical College Hospital, Chinese Academy of Medical Science and Peking Union Medical College, Beijing 100730, China

## Abstract

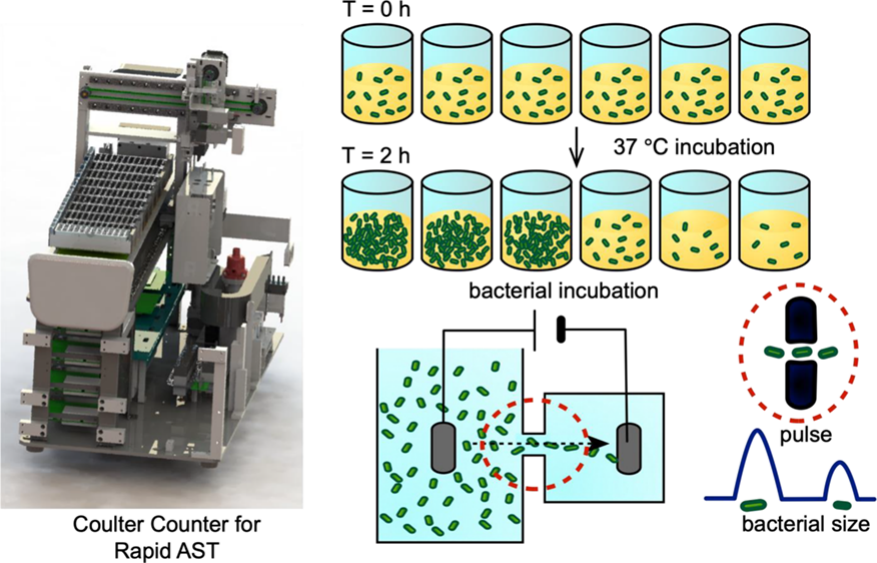

The rapid determination of antimicrobial susceptibility and evidence-based antimicrobial prescription is necessary to combat widespread antimicrobial resistance and promote effectively treatment for bacterial infections. This study developed a rapid phenotypic antimicrobial susceptibility determination method competent for seamless clinical implementation. A laboratory-friendly Coulter counter-based antimicrobial susceptibility testing (CAST) was developed and integrated with bacterial incubation, population growth monitoring, and result analysis to quantitatively detect differences in bacterial growth between resistant and susceptible strains following a 2 h exposure to antimicrobial agents. The distinct proliferation rates of the different strains enabled the rapid determination of their antimicrobial susceptibility phenotypes. We evaluated the performance efficacy of CAST for 74 clinically isolated *Enterobacteriaceae* subjected to 15 antimicrobials. The results were consistent with those obtained via the 24 h broth microdilution method, showing 90.18% absolute categorical agreement.

## Introduction

The growing risk of infection by multidrug-resistant pathogens^1^ and increasing incidence of clinical treatment failure are primarily occurring due to the misuse of antibiotics, which induces resistant phenotype development in pathogenic organisms.^2^ Improving antimicrobial prescription and providing evidence-based treatments in clinical practice are essential for effectively treating infections, protecting patients from harm caused by unnecessary antibiotic use, and combating antimicrobial resistance.^3^ However, once specimens, such as infected blood, are collected and delivered to a laboratory for antimicrobial susceptibility testing (AST), the results are typically available only after the 3–5 days required for conventional diagnostic testing. In the interim, improper and unnecessary treatment is potentially administered.^4^ Thus, rapid diagnostics can help guide antimicrobial prescription in a timely fashion and can be a critical determinant of patient outcomes.^5^

Rather than molecular AST,^5^ culture-based methods will remain irreplaceable until a new method is found that satisfies two diagnostic requirements: assisting clinicians to rapidly select appropriate antimicrobial treatments for bacterial infections and monitoring epidemiological changes.^1^ Applicable sample-to-answer methods using pheno-molecular AST platforms are mainly limited to urine specimens.^6,7^ Recently, there has been an emergence of rapid AST-based methods, especially phenotypic AST.^8−11^ The arrest of bacterial growth in the presence of antimicrobials was achieved by metabolic activity detection,^12,13^ morphologic change,^14,15^ and gene expression,^16^ using labeling methods, such as fluorescence resonance energy transfer probes,^17^ D_2_O-containing media,^8^ and hybridization probes.^7,18^ The most direct manifestation of antimicrobials is killing or inhibiting the growth of microorganisms. Once the antimicrobial is administered, antimicrobial activity includes bacteriostatic or bactericidal action. Following antimicrobial administration, the growth of susceptible strains is considerably inhibited compared with that of resistant strains. Strategies to observe microbial growth or inhibition longitudinally focus on determining increases, decreases, or equilibrium in the number of bacterial cells. Bacterial cell enumeration methods involve the detection of bacterial cell division at an early stage,^11^ fluorescence signaling of metabolic activity of bacterial cells,^12^ and tracking single cell growth via morphological analysis.^15,19^ Gravity-driven microfluidic devices^20^ and cell-trapping microchannels^15^ have also been used to precisely enumerate the number of bacterial cells.

Among those, flow cytometry offers absolute bacterial cell enumeration in a high-throughput way and considerably decreases antimicrobial susceptibility testing time-to-result.^21^ However, fluorescent dyes play an essential role in classical flow cytometry,^22^ and the flow cytometric measurement of single-cell sizes involves dyes that adhere to intracellular molecules and surface antigens and rely heavily on fluorescence labeling for cellular phenotyping.^23^ Electronic sensors, such as Coulter counters and impedance cytometers, characterize particles via resistive-pulse sensing and are accepted as label-free, noninvasive, and quantitative analytical tools for assessing cell status.^24−26^ The impedance-based fast antimicrobial susceptibility test (iFAST) using microfluidics can deliver a resistance profile in 30 min.^27^ Furthermore, a digital electrical impedance sensing platform enables the direct multiplex measurement of single bacterial cells.^28^ However, a versatile and adaptable AST technology for routine clinical analysis is not yet available. In addition, practical considerations of cost and staff training should be accounted for in clinical microbiology laboratories. Thus, a ready-to-use rapid AST system that acts as a substitute for conventional methods in clinical laboratories should be able to assess specimens received in bulk and respond to specific requests using an automated process regardless of infection type.

**Figure 1:**
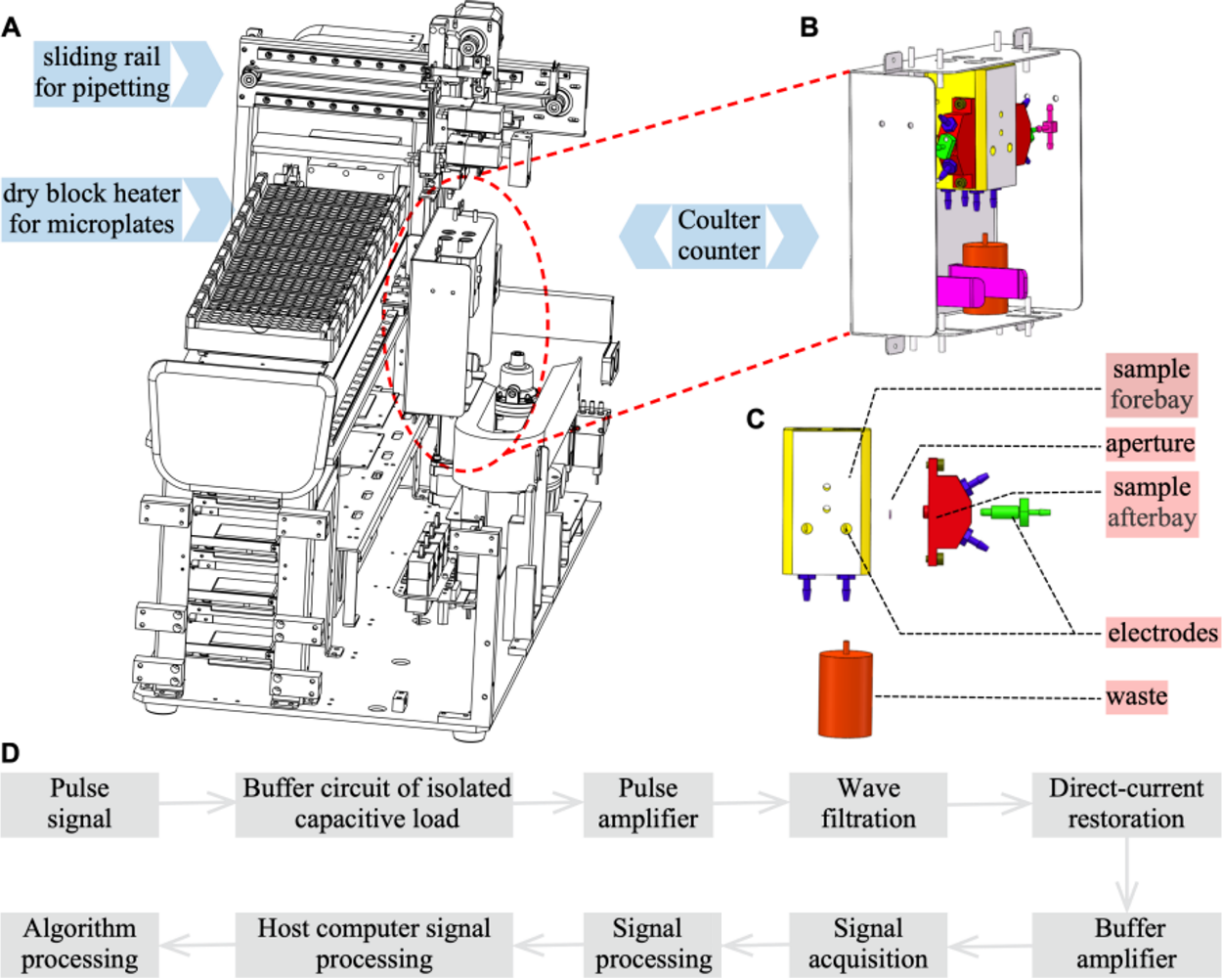
Schematic of the Coulter counter used exclusively for antimicrobial susceptibility testing. (A) Sketch and development of the CAST, including the functions of bacterial incubation, population enumeration, and result interpretation. (B) Part of the Coulter counter for bacterial enumeration. (C) Setup of the Coulter counter. (D) General signal processing process of the system.

Here, we developed a Coulter counter exclusively for AST that combines bacterial incubation, population growth monitoring, and result interpretation (Figure 1). This Coulter counter-based AST (CAST) system enumerates the number of bacterial cells based on the Coulter principle. Compared with other methods, this assay has the advantages of (i) producing phenotypic AST results after a 2-h exposure to an antimicrobial, (ii) being able to count all types of cells without labeling, (iii) practical implementation by users of the current AST methods, and (iv) eliminating the problem of device-to-device variation in microfluidics. We produced antimicrobial susceptibility phenotype profiles of bacterial strains by detecting electrical impedance. Enumerating the number of bacterial cells in the presence of an antimicrobial enables the rapid determination of the pathogens’ susceptibility.

Gram-negative bacteria are responsible for the majority of conspicuous morbidity and mortality worldwide.^29^ In addition, Gram-negative bacteria are more resistant than Gram-positive bacteria owing to the distinctive structure of the former.^30^ The CAST described herein was validated with commonly isolated Gram-negative bacteria and evaluated for bacterial growth and susceptibility determination using 74 clinical isolates. The results were consistent with those obtained using the Clinical and Laboratory Standards Institute (CLSI) recommended reference method, broth microdilution method (BMD).^31^

## Results and Discussion

### Working Principle of Bacterial Cell Counting and Verification

**Figure 2:**
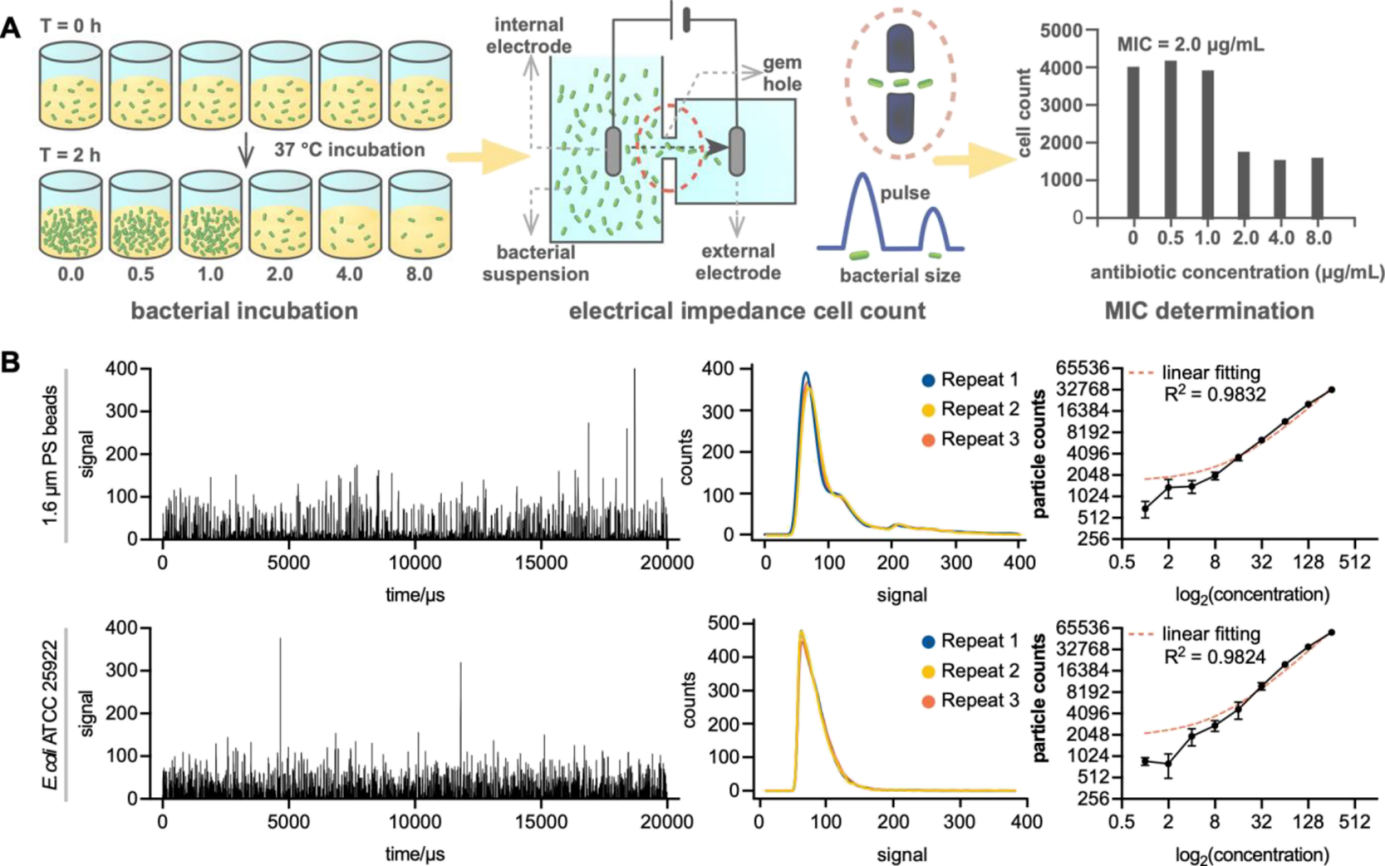
Working principle of CAST and verification of bacterial counting. (A) Scheme for antimicrobial determination. Bacteria were incubated for 2 h under various concentrations of antimicrobials, and then, the number of bacteria was counted via the Coulter counter. Minimum inhibitory concentration was determined according to the difference in cell count. (B) CAST results for 1.6 μm beads and *E. coli* through the 50 μm gem hole. A typical time series recording of the detected electric signal of particles transit (left). Histogram showing the distribution of particle sizes for each particle with three repeats (middle). Linear correlation between concentration and particle counts (right). The initial concentrations for PS beads and *E. coli* were 10^7^ particles/mL and 10^8^ CFU/mL, respectively.

A Coulter counter is primarily used to count blood cells in clinical practice. The principle behind counting particles is based on current disruption in a chamber due to a nonconductive particle as it passes through the electric field and displaces its own volume of electrolyte. Such a current disruption is recorded as one particle. Figure 1 shows the schematic of the Coulter counter used exclusively for antimicrobial susceptibility testing, and expounded on how the system works. As shown in the conceptual graph (Figure 2A), the CAST system is exclusively built to identify the antimicrobial susceptibility phenotypes of bacterial strains after a 2 h antimicrobial exposure. We used the Coulter counter to directly enumerate the number of bacterial cells in serial dilutions. Hence, to demonstrate that the system is adequate for determining bacterial antimicrobial resistance, we needed to answer the following question: can the method appropriately enumerate the number of bacterial cells and distinguish between resistant and susceptible bacteria in real-world settings?

The Coulter counter prescribes a limit to the size range of particles that can be measured. Hence, the prerequisite for enumerating bacterial cells is that the bacterial size is sufficient to be detected. *Escherichia coli* ATCC 25922 and similar-sized 1.6-μm monosized polystyrene (PS) beads were used to verify the cell counting ability of CAST (Figure 2B). With the passage of each particle through the aperture, the resistance of the electrical path between the submerged electrodes on each side of the aperture momentarily increased, and the measurable resistive-pulse was recorded (Figure 2B, left). The impedance signal is proportional to particle size,^32^ and a current and frequency of 5 mA and 1 MHz, respectively, were applied to the electrodes. Pulse data or impedance magnitude, determined by the amplitude of the differential voltage signals acquired against the baseline, is presented in arbitrary units to enable comparisons across samples. The number of pulses and their amplitude enable the measurement and analysis of parameters, including the number of bacterial cells. The pulse data were converted to size distributions (Figure 2B, middle).

For a conventional OD/turbidity–based AST method, the bacterial cell concentration begins from ∼10^5^, consequently increasing to a detectable concentration of ∼10^7^ CFU/mL.^33^ The limited sensitivity of the lower concentrations of bacterial cells forces an incubation of 18–24 h to reach detectable levels. Therefore, determinations are only possible when considerable differences appear between initial and final states. To ensure that the results of the different methodologies remain comparable, dilutions of the suspensions containing only PS beads or *E. coli* ATCC 25922 cells were prepared from the initial to OD-detectable concentrations (10^5^–10^7^ particles/mL).

Theoretically, the recorded bacterial cell counts should be linearly correlated with the concentrations of the suspensions. Such correlations were observed for nine two-fold dilutions corresponding to bacterial binary fission, demonstrating good linear correlation between bacterial cell concentration and particle counts. Similar results were obtained using 1.6 μm PS beads and *E. coli* ATCC 25922 cells (Figure 2B, right).

### Verification of CAST with Various Bacterial Species

Most pathogenic bacteria rely on binary fission for propagation, wherein a single bacterium split into two. Hence, fold changes in bacterial cell counts reflect bacterial growth. However, the doubling time of a bacterium varies from species to species. Thus, an additional experiment was performed to assess the suitability of CAST to characterize commonly encountered pathogens; these data were used to set a rational duration for the method. The bacterial cell counts over time obtained via CAST were used to monitor dynamic changes in the cell growth of 10 commonly used species in clinical practice, including *E. coli* (eco), *Klebsiella pneumoniae* (kpn), *Pseudomonas aeruginosa* (pae), *Acinetobacter baumannii* (aba), *Enterobacter cloacae* (ecl), *Serratia marcescens* (sma), *Proteus mirabilis* (pmi), *Morganella morganii* (mmo), *Burkholderia cepacia* (bce), and *Salmonella enterica* (sen). Bacteria were incubated for 4 h, and their growth was measured every 30 min. The initial bacterial suspensions were 1000-fold dilutions from a 0.5 McFarland bacterial culture. To account for variations in the initial bacterial count of the different species, the increases in cell counts were normalized to the initial count. The instantaneous fold change of the bacterial cell counts at each time point was calculated for better comparability.

**Figure 3:**
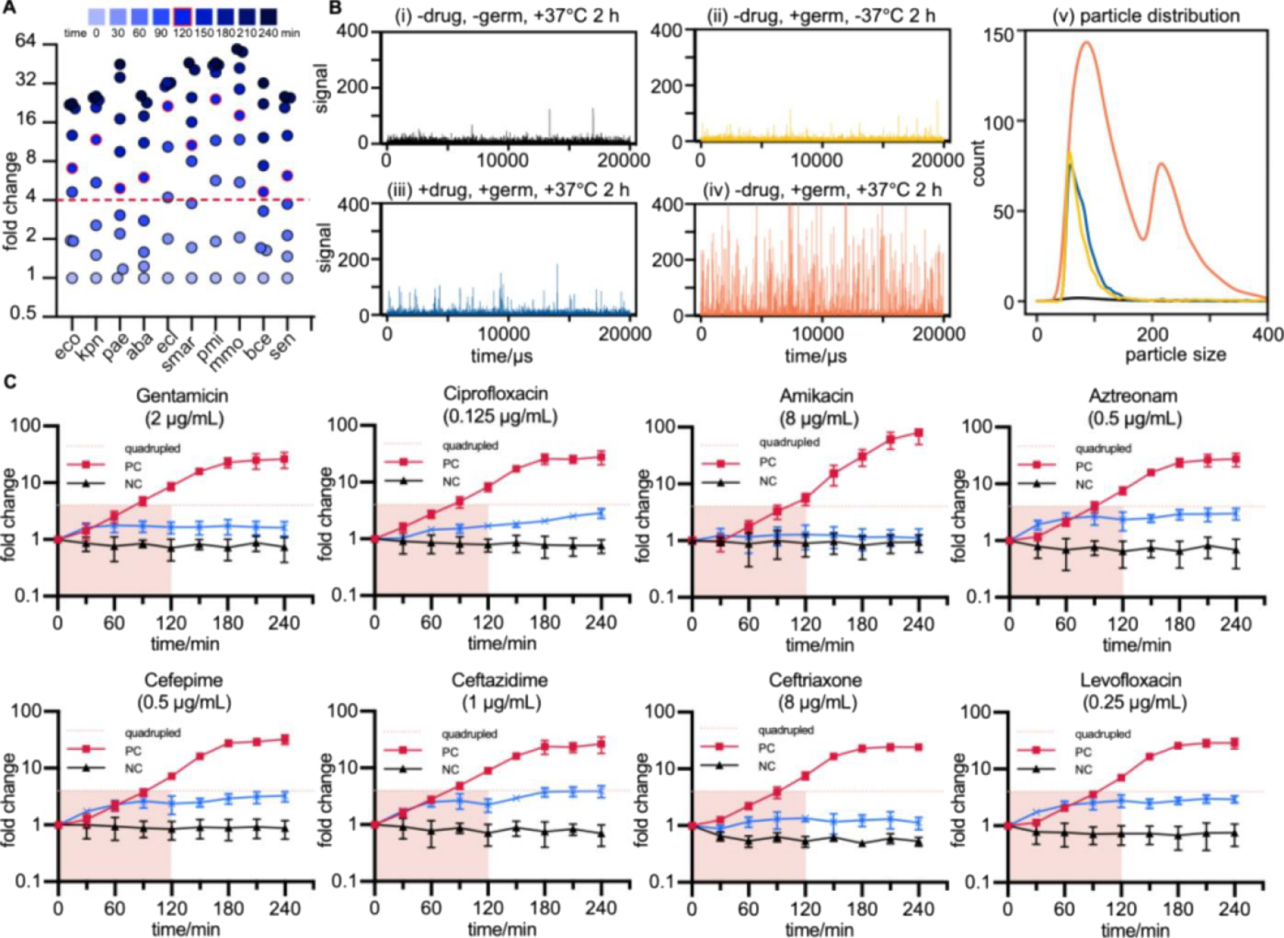
Tests of CAST compatibility with various bacterial species and antimicrobial susceptibility determination. (A) Average bacterial enumeration fold change was collected every 30 min from the initial concentration for 10 stains, including *Escherichia coli* (eco), *Klebsiella pneumoniae* (kpn), *Pseudomonas aeruginosa* (pae), *Acinetobacter baumannii* (aba), *Enterobacter cloacae* (ecl), *Serratia marcescens* (smar), *Proteus mirabilis* (pmi), *Morganella morganii* (mmo), *Burkholderia cepacia* (bce), and *Salmonella enterica* (sen). The red contour shows the fourfold change in 120 min. (B) CAST detection of *E. coli* cells suspended in CAMHB after incubation for 2 h (i) without antimicrobials and bacteria, (ii) without antimicrobials and incubation, (iii) with 2 μg/mL gentamicin, and (iv) without antimicrobial. (v) Particle distribution for (i–iv). (C) Quantitative measurement of the bacterial population for *E. coli* ATCC 29522, a quality control strain for AST, cocultured with a wide range of different antimicrobial agents at breakpoint concentration (blue line). Suspensions without antimicrobials were set as the positive control (red line), and suspensions without bacteria were set as the negative control (black line).

After comparing with the initial bacterial cell counts, the bacterial growth profile of each species (three repeats/strain) was obtained. The kinetics of the growth of each species were analyzed to rapidly determine their proliferation rates. We considered an approximately 2^4^-fold increase in bacterial cell counts as normal growth. Changes of this magnitude were observed within 2 h (Figure 3A). Hence, the duration of AST was set as 2 h. This timeframe produced robust results in the calibration experiments, unless otherwise specified, this incubation time was used throughout the study.

### Performance of CAST in Antimicrobial Resistance Determination

CAST rapidly assessed bacterial antimicrobial susceptibility via the quantitative detection of bacterial counts. Antimicrobials of different concentrations were incubated with bacteria, and the cell number of bacteria was enumerated over a period of incubation. The counted number was calculated to assess the antimicrobial effects on the bacteria. As the bacterial cell number changes at different antimicrobial doses, clinically relevant minimum inhibitory concentration (MIC) was determined. To demonstrate this, four groups of *E. coli* ATCC 25922 cells suspended in cation-adjusted Mueller–Hinton broth (CAMHB) were measured (Figure 3B): (i) only CAMHB was incubated for 2 h without antimicrobials and bacteria, as the blank control (−drug, −germ, +37 °C); (ii) the bacterial suspension was tested at the initial state without incubation and antimicrobials (−drug, +germ, −37 °C); (iii) the bacterial suspension was incubated at 37 °C for 2 h with 2 μg/mL gentamicin (+drug, +germ, +37 °C); and (iv) the bacterial suspension was incubated at 37 °C for 2 h without an antimicrobial (−drug, +germ, +37 °C). The minimum inhibitory concentration of gentamicin for *E. coli* ATCC 25922 was 0.25–1 μg/mL, and 2 μg/mL gentamicin exhibited bactericidal effects. Each set of suspensions was assessed using CAST, and the results were presented in a histogram. The negative control (only CAMHB, Figure 3B-i), culture-free (Figure 3B-ii), and cultured *E. coli* cells with and without gentamicin (Figure 3B-iii,iv) exhibited total bacterial counts of 412, 4732, 41,148, and 5292, respectively, consistent with the expected 2^3^–2^4^-fold increase based on the initial concentrations of *E. coli*. CAST indicated that the spikes of cells passing through the 50 μm aperture were distinctly different between the resistant and susceptible bacteria (Figure 3B).

As the effects of different antimicrobials on bacteria are heterogeneous,^33,34^ various antimicrobial agents were assessed using CAST. The growth curve of *E. coli* ATCC 29522, a quality control strain for AST, with and without antimicrobials at inhibited concentration was assessed using CAST (Figure 3C). *E. coli* exposed to antimicrobials at the inhibitory boundary (blue line) were compared with the positive controls (bacterial suspension without antimicrobials, red line) and negative controls (culture media without antimicrobials and bacteria, black line). The antimicrobial concentrations, gentamicin 2 μg/mL, ciprofloxacin 0.125 μg/mL, amikacin 8 μg/mL, aztreonam 0.5 μg/mL, cefepime 0.5 μg/mL, ceftazidime 1 μg/mL, ceftriaxone 8 μg/mL, and levofloxacin 0.25 μg/mL, were selected to ensure that the bacterial growth at lower concentrations was too low to influence the proliferation rate based on the CLSI guidelines. The bacterial growth assessed every 30 min revealed that the number of bacterial cells did not increase above the 2^4^-fold threshold in the presence of antimicrobial at inhibitory concentrations and that the antimicrobial effects on bacteria can be differentiated by comparing fold changes in bacterial cell counts within 2 h. Thus, it is reasonable to define a 2^4^-fold increase as a threshold independent of the antimicrobials category.

### Minimum Inhibitory Concentrations and Breakpoint Analysis for Clinical *Enterobacteriaceae* Isolates

The MIC was obtained, and breakpoint analysis was conducted for clinical *Enterobacteriaceae* isolates in a real-world clinical microbiology laboratory. In total, 74 isolates, including 38 *E. coli*, 17 *K. pneumoniae*, 5 *E. cloacae*, 4 *S. marcescens*, 4 *P. mirabilis*, 2 *Salmonella* spp., 2 *E. aerogenes*, 1 *M. morganii*, and 1 *P. stuartii* (Table 1) strains, were analyzed using CAST, and the results were compared with those obtained from the reference method, broth micro-dilution (BMD). These strains were exposed to 15 antimicrobial agents, including ampicillin, aztreonam, trimethoprim–sulfamethoxazole (SXT), ciprofloxacin, meropenem, gentamycin, tetracycline, cefepime, ceftazidime, cefoxitin, cefazolin, tobramycin, imipenem, colistin, and levofloxacin, with each of these antimicrobials having clinical indications that should be considered for routine testing and reporting of *Enterobacteriaceae*. The duration of CAST was decided as 2 h, and a 2^4^-fold increase from the initial bacterial cell count was considered the demarcation of antimicrobial susceptible and resistant. That means, resistance was defined as a ≥2^4^-fold increase in bacterial cell counts following incubation for >2 h.The breakpoint interpretive standards, resistant (R), susceptible (S), and intermediate (I), for *Enterobacteriaceae* from the 30th CLSI M100 were used. A confusion matrix for binary classification was used for comparison with the reference method. The accuracy, sensitivity, and specificity of CAST in identifying resistance were 93.33, 89.42, and 97.91%, respectively. The accuracy, sensitivity, and specificity of CAST in identifying susceptibility were 93.42, 97.24, and 91.29%, respectively. In clinical practice, when implementing a new AST method, acceptability for verification testing is >90% of the categorical agreement (CA) for S/I/R identification compared with that of the reference method. Herein, for all the 1100 tests, the absolute CA for the isolates was 90.18%, with 30 very major errors (VME, susceptibility, and resistance results via the new and reference methods, respectively), eight major errors (ME, resistance, and susceptibility via the new and reference methods, respectively), and 71 minor errors (MIE, susceptibility, and intermediate via the new and reference methods, respectively, or intermediate and susceptibility via the new and reference methods, respectively). The frequency of VME, ME, and MIE of CAST compared with that of the BMD method was the highest for ceftazidime (10.81, 0, and 13.51%, respectively) followed by imipenem (8.11, 0, and 9.46%, respectively). Conversely, no VME was observed for SXT, cefazolin, and colistin.

**Table 1:** Comparison between the CAST and the Reference Microbroth Dilution Method in Testing the Susceptibility of 74 *Enterobacteriaceae* Isolates to 15 Antimicrobials

antimicrobials	I^b^	R^b^	S^b^	CA%^b^	ME%^b^	MIE%^b^	VME%^b^
ampicillin	1	71	2	95.95	0.00	2.70	1.35
aztreonam	2	45	27	90.54	2.70	4.05	2.70
SXT	0	49	25	98.65	1.35	0.00	0.00
ciprofloxacin	8	55	11	93.24	0.00	5.41	1.35
meropenem	1	12	61	93.24	2.70	1.35	2.70
gentamycin	2	33	39	93.24	2.70	2.70	1.35
tetracycline	5	48	21	90.54	0.00	8.11	1.35
cefepime	10^a^	38	26	79.73	0.00	17.57	2.70
ceftazidime	5	46	23	75.68	0.00	13.51	10.81
cefoxitin	1	28	45	91.89	1.35	2.70	4.05
cefazolin	6	64	4	91.89	0.00	8.11	0.00
tobramycin	6	22	46	89.19	0.00	9.46	1.35
imipenem	6	15	53	82.43	0.00	9.46	8.11
colistin	62	12	0	95.95	0.00	4.05	0.00
levofloxacin	11	48	15	90.54	0.00	6.76	2.70
total	126	586	398	90.18	10.81	95.95	40.54

a Note: Cefepime has a new category for antibacterial susceptibility testing, susceptible-dose dependent (SDD). Here for better comparison, SDD is categorized as intermediate (I).

b R, resistant; S, susceptible; I, intermediate. CA, categorical agreement; VME, very major errors; ME, major errors, MIE, minor errors.

The limitation of the proposed method includes validation with gram-negative bacteria only. Thus, the utility of CAST for Gram-positive bacteria remains unknown. In our subsequent research, we will focus on improving the sensitivity of CAST to enable the assessment of Gram-positive bacteria, including *Staphylococcus* and *Enterococcus*. Furthermore, by analyzing the relation between the impedance signal intensity and bacteria size, it is theoretically to count the number of bacteria of different sizes, as well as blood cells. AST using colonies is universal for all specimens for isolating and enriching pathogens, which contradicts its use for rapid assessment. If rapid tests are performed directly using raw specimens, such as blood or positive blood cultures, regardless of the infection location, a better solution can be achieved to accelerate clinical diagnosis.

## Conclusions

Herein, we used a Coulter counter to analyze bacterial susceptibility to antimicrobials for clinical application. The results of CAST exhibited excellent consistency with those obtained using the conventional BMD method. In addition, a 2-h incubation was confirmed as sufficient for determining bacterial resistance. Growth inhibition (susceptibility) was distinguished based on bacterial counts, allowing for the calculation of MICs. Thus, using CAST involving serial dilutions to obtain MIC, which is familiar to medical laboratory technicians and does not require substantial changes to operations rather than a new technology, is more feasible. Therefore, a laboratory-friendly, high-throughput rapid testing system with practical, affordable, and automated operation would help clinicians prescribe appropriate antimicrobial agents to treat infectious diseases and promote diagnostic stewardship.

## Materials and Methods

### Bead Preparation

PS beads 1.6 μm in diameter (Duke Standards NIST traceable Polymer Microspheres, Cat. No 4016A, Thermo Fisher Scientific) and *E. coli* ATCC 25922 were used as model particles. The initial concentration of the 1.6-μm beads was 10^7^ particle/mL, a 100-fold dilution from the original commercial suspension. The initial concentration of *E. coli* ATCC 25922 was 0.5 McFarland (equal to 1 × 10^8^ CFU/mL). Each suspension was serially diluted with saline to obtain nine bacterial and particle suspensions, 1×, 1/2×, 1/4×, 1/8×, 1/32×, 1/64×, 1/128×, 1/256×, and 1/512×. The diluents were sieved through 0.22 μm filters. The impedance signals from the Coulter counter were measured at a frequency of 1 MHz.

### Bacterial Suspension Preparation

The bacterial suspensions prepared for AST used *E. coli* ATCC 25922 as a control for the antimicrobial susceptibility tests. Other bacterial strains were obtained from the Clinical Laboratory of Peking Union Medical College Hospital (Beijing, China) and Liaocheng People’s Hospital (Shandong, China). Each strain was streaked on blood agar and incubated at 37 °C overnight. A single normal-appearing colony was picked and inoculated into filtered CAMHB. The suspension was subsequently adjusted to the 0.5 McFarland Standard as recommended by the CLSI.^31^

### Antimicrobial Susceptibility Testing

The AST panels were preloaded with antimicrobials via freeze-drying. The AST assays used 300 μL 0.5 McFarland bacterial suspension diluted to 30 mL CAMHB. Then, 500 μL of diluted bacterial suspension was added to the wells in paired plates and mixed. The contents of the wells in one plate were assessed using CAST to determine the susceptibility and resistance profiles following incubation at 37 °C for 2 h. For comparison, the paired wells from the second plate were assessed using the conventional BMD method following incubation for 18–20 h. The susceptibility and resistance profiles of the bacterial species were determined based on visible growth, according to the CLSI criteria. The BMD method was used as a gold standard as recommended by the CLSI.

### Continuous-Flow Bacterial Counting

Continuous-flow bacterial counting was performed using the Coulter counter (Figure 1). A direct current (DC) excitation signal at a frequency of 1 MHz was applied to platinum electrodes alongside the flow channel. The bacterial suspensions were incubated on a dry block heater for culture plates. Before each count, the counting chamber was washed and cleaned. When counting, an aspirating needle drew 360 μL of liquid from each well into the front pool, and this aliquot was simultaneously mixed with 2200 μL of diluent. A plunger pump (Juray Electrical Technology Co., Ltd., Dongguan, China) with a negative pressure of −30 kPa and a flow rate of 6 m/s were used to push liquid suspension through the counter. The size of the gem hole was 50 μm (I.D.). Voltage pulses occurred and were recorded when single cells increased resistance. The bacterial cell counts in the suspensions were proportional to the number of pulses, and differences among the cell counts distinguished antimicrobial-resistant and -susceptible strains. For each test, signal acquisition continued for 8 s. In all data presentations, impedance magnitude was normalized to the amplitude of the voltage signal acquired against the baseline and was described in arbitrary units to enable comparison across samples.

### Statistical Analysis

Statistical analysis was conducted using GraphPad Prism version 9.0 (GraphPad Software Inc.) and R version 4.1.1 (R Project for Statistical Computing) with the R package (ggplot). The signal acquisition frequency of the instrument was 1 MHz, and every cell count collected 5,000,000 signals, which were divided into 250 groups. Each group thus represented 20,000 signals. The peaks were analyzed after eliminating the baseline and signals of >400. The final cell counts were enumerated at signals between 50 and 306. The impedance data collected were analyzed in C language and are available from the corresponding author on reasonable request. Statistical analysis of the diagnostic test results, including accuracy, sensitivity, and specificity, was conducted using a matrix for binary classification, revealing four different outcomes: true positive (TP), false positive (FP), true negative (TN), and false negative (FN). In detail, accuracy = (TP + TN)/(TP + FP + FN + TN) × 100%, sensitivity = TP/(TP + FN), and specificity = FP/(FP + TN).

## Notes

The data supporting this study’s findings are available upon request from the corresponding author.
